# Clone- and age-dependent toxicity of a glyphosate commercial formulation and its active ingredient in *Daphnia magna*

**DOI:** 10.1007/s10646-012-1021-1

**Published:** 2012-12-06

**Authors:** Marek Cuhra, Terje Traavik, Thomas Bøhn

**Affiliations:** 1 GenØk, Centre for Biosafety, The Science Park, P.O. Box 6418, 9294 Tromsø, Norway; 2 Faculty of Health Sciences, University of Tromsø, Tromsø, Norway

**Keywords:** Cladocera, Glyphosate, Roundup, Aquatic, Chronic toxicity, Toxicology

## Abstract

Low levels of glyphosate based herbicide induced significant negative effects on the aquatic invertebrate *Daphnia magna.* Glyphosate herbicides such as brands of Roundup, are known to be toxic to daphnids. However, published findings on acute toxicity show significant discrepancies and variation across several orders of magnitude. To test the acute effects of both glyphosate and a commercial formulation of Roundup (hereafter Roundup), we conducted a series of exposure experiments with different clones and age-classes of *D. magna*. The results demonstrated EC_50_ (48) values in the low ppm-range for Roundup as well as for the active ingredient (a.i.) isopropylamine salt of glyphosate (glyphosate IPA) alone. Roundup showed slightly lower acute toxicity than glyphosate IPA alone, i.e. EC_50_ values of 3.7–10.6 mg a.i./l, as compared to 1.4–7.2 mg a.i./l for glyphosate IPA. However, in chronic toxicity tests spanning the whole life-cycle, Roundup was more toxic. *D. magna* was exposed to sublethal nominal concentrations of 0.05, 0.15, 0.45, 1.35 and 4.05 mg a.i./l for 55 days. Significant reduction of juvenile size was observed even in the lowest test concentrations of 0.05 mg a.i./l, for both glyphosate and Roundup. At 0.45 mg a.i./l, growth, fecundity and abortion rate was affected, but only in animals exposed to Roundup. At 1.35 and 4.05 mg a.i./l of both glyphosate and Roundup, significant negative effects were seen on most tested parameters, including mortality. *D. magna* was adversely affected by a near 100 % abortion rate of eggs and embryonic stages at 1.35 mg a.i./l of Roundup. The results indicate that aquatic invertebrate ecology can be adversely affected by relevant ambient concentrations of this major herbicide. We conclude that glyphosate and Roundup toxicity to aquatic invertebrates have been underestimated and that current European Commission and US EPA toxicity classification of these chemicals need to be revised.

## Introduction

The tonnage of glyphosate herbicide application has been constantly increasing since the introduction of this group of chemicals in 1971 (Dill et al. 2010). The 2008 global production was estimated to be 620.000 tonnes, representing a value of 8.3 billion US$, making glyphosate the most widely used herbicide ingredient worldwide (Pollak 2011). The most common herbicide formulations such as the brands of Roundup contain various salts of glyphosate that ensure high water solubility, mainly isopropylamine salt (IPA salt of glyphosate) (Woodburn 2000). Introduction of glyphosate tolerant transgenic crops such as ‘Roundup-Ready’ soy, maize, canola, sugarbeet and cotton, contribute to a further rapid expansion of use (Antoniou et al. 2010, Cerdeira and Duke 2006). Nearly 90 million hectares were planted with herbicide tolerant GM plants in 2010, primarily with glyphosate tolerant traits and primarily in North- and South America (James 2010). Glyphosate has been heralded as an ideal herbicide due to its target specificity and acclaimed low toxicity to non-target organisms (Cerdeira and Duke 2006; Duke and Powles 2008; Giesy et al. 2000). The majority of glyphosate herbicide applications, measured in gross tonnage as well as number of herbicide brands, are in agriculture. Some use is also related to forestry, gardening and park management and even specialized applications such as weed management in fresh water lakes and streams (Simenstad et al. 1996). Field studies on effects of prescription dosage application of aquatic glyphosate herbicide on non-target organisms in fresh-water systems have been carried out. Most of these have neither demonstrated short-term nor long term adverse effects (Gardner and Grue 1996, Siemering et al. 2008), although Puértolas et al. (2010) observed significant toxic effects on the aquatic invertebrate *Daphnia magna* from established methods for glyphosate control of giant reed in a Spanish River system.

Some field studies on effects of glyphosate herbicides in daphnids have not shown adverse effects in modelled instances of herbicide drift from agriculture bordering wetlands (Hessen et al. 1994), even when using dosages much higher (×10 and ×100) than prescribed for agriculture use (Hildebrand et al. 1980). However, high levels of glyphosate have been measured in streams draining agricultural fields of transgenic ‘Roundup Ready’ soybeans, with adverse effects on non-target aquatic biodiversity (Ronco et al. 2008).

Dynamics of glyphosate in soil, water and sediment have been well studied and its presence has been reported in general surface waters (Scribner et al. 2007; Struger et al. 2008; USGS 2010) as well as in farmland streams (Peruzzo et al. 2008, Ronco et al. 2008). We have reviewed the literature for short- and long-term toxicity studies of glyphosate and glyphosate based herbicides in aquatic organisms. This literature is based mainly on laboratory experiments, with some evidence derived from mesocosm-studies and field-studies. Some studies of effects on non-target organisms indicate that glyphosate herbicides in fresh-water and marine ecosystems can have significant negative effects on for instance aquatic microbial communities (Pérez et al. 2007), macrophytes (Lockhart et al. 1989; Simenstad et al. 1996), cnidaria (Demetrio et al. 2012), sea-urchin embryogenesis (Marc et al. 2004), fish (Servizi et al. 1987), amphibians (Mann et al. 2009; Relyea 2005) and planktonic algae (Peterson et al. 1994; Pérez et al. 2007). However, a majority of relevant publications report low toxicity or no adverse effects from prescribed dosage use. This also corresponds to conclusions in published reviews of glyphosate-based herbicide ecotoxicity potential (Giesy et al. 2000; Dill et al. 2010). A recent review of glyphosate herbicide effects in aquatic ecosystems gives a comprehensive overview of individual studies for most investigated taxonomic groups (Pérez et al. 2012).

Laboratory studies testing ecotoxicological effects of glyphosate and various glyphosate-based herbicides on specific aquatic organisms have been performed for four decades, with varying results even in the same test-species. Acute (immediate, short-term) glyphosate toxicity to aquatic invertebrates such as the model organism *D. magna* is generally considered by regulators to be relatively low (EC 2002; US EPA 1993; Mensink and Janssen 1994). Baseline effect studies and toxicological testing establishing EC_50_ and LC_50_ treshold values for glyphosate and glyphosate formulations in *D. magna* and other species of daphnids have shown highly variable results, ranking these chemicals from practically non-toxic to moderately toxic (FAO 2001; Folmar et al. 1979; McAllister and Forbis 1978; Melnichuk et al. 2007a; Tsui and Chu 2003). To some extent these differences have been attributed to additive or synergistic effects of non-specified “inert ingredients” in herbicide formulations (Folmar et al. 1979; Melnichuk et al. 2007a) including adjuvants and additives, such as Polyethoxylated tallowamine (POEA) used for dispersal and increased plant uptake (Brausch et al. 2007). A study comparing acute toxicity of 6 different brands of formulated glyphosate-based herbicides in *D. magna* found LC_50_ values in the range 4.2–117 mg/l and highlighted the fact that published literature presents EC_50_ values spanning 3 orders of magnitude (Melnichuk et al. 2007a). Even ecotoxicological testing of the active ingredient glyphosate alone has given highly divergent results in *D. magna*. Some studies have reported LC_50_ values of 13–24 mg/l (FAO 2001) whereas others report values of 234 mg/l (Le et al. 2010), 780 mg/l (McAllister and Forbis 1978), 930 mg/l (Forbis and Boudreau 1981) or even above 2000 mg/l (Pereira et al. 2009).

Long-term (chronic) exposure studies of glyphosate in *D. magna* comissioned by the producing industry have shown NOEC (no observed effect concentration) of 50 mg/l for glyphosate-IPA salt (McKee et al. 1982) but later independent studies of formulated glyphosate-based herbicides have shown substantially higher chronic toxicity in *D. magna*, with effects in concentrations of 2, 0.2 and even 0.02 mg/l glyphosate as active ingredient (a.i.) in a Roundup formulation (Papchenkova 2007).

The inconsistent results with regard to toxicity of glyphosate and glyphosate-based herbicides may suggest varying sensitivity between different clones of *D. magna*. The literature lends some support for clone-specific sensitivity to metals and organic toxins like cadmium, copper, dichloraniline and benzyl sulfonate (Baird et al. 1991).

In the present work we tested the following hypotheses: (i) acute toxicity (EC_50_) of glyphosate and a commercial Roundup formulation differs between clones of *D. magna* (clone-specific toxicity); (ii) acute toxicity of glyphosate and Roundup decreases with increasing age in *D. magna* (age-specific toxicity); (iii) chronic exposure to glyphosate and Roundup induces adverse effects on *D. magna* life-history traits (survival, growth, fecundity, abortion rate and juvenile body size) at much lower concentrations than acute EC_50_-values; (iv) Roundup is more toxic than glyphosate at the same concentration of active ingredient since Roundup contains additional potentially toxic chemicals.

## Materials and methods

Clones of *D. magna* were obtained from laboratories at the Universities of Oslo (Tromsø-clone, courtesy of Dag Hessen, Dept. of Biology, University of Oslo, P.O. Box 1066 Blindern, NO-0316 Oslo, Norway), Reading (Reading-clone, courtesy of Richard Sibly, Amanda Callaghan, Chris Hill, School of Biological Sciences, The University of Reading, Whiteknights, PO Box 217, READING, Berkshire, RG6 6AH, United Kingdom) and Leuven (Knokke-clones, courtesy of Luc De Meester and Sarah Rousseaux (Katholieke Universiteit Leuven, Laboratory of Aquatic Ecology and Evolutionary Biology, Charles Deberiotstraat 32—box 2439, 3000 Leuven). These clones have very different backgrounds, as some are wild clones collected in Belgium from pristine lakes (Knokke 1 = KNO-15 NF14 and Knokke 2 = KNO-15-F5), from “extensive agriculture intermediate ponds”, where some anthropogenic chemicals are expected (Knokke 3 = KNO 208 F1) and from ponds in areas of intensive agriculture (Knokke 4 = KNO-16-F8) (nomenclature of Luc De Meester, Coors et al. 2009). Two clones are from cultures reared for years in laboratories (clones Tromsø and Reading). Taxonomic control of clones (species) was performed according to defined morphological characters (Benzie 2005).

Aqueous solution of 40 % b.w. glyphosate in the form of N-(phosphonomethyl) glycine-monoisopropylamine salt (glyphosate-IPA), hereafter ‘glyphosate’ in this work, was obtained from Sigma–Aldrich, St. Louis MO 63103 USA (Batch no 10519EJ). A typical commercial brand of Roundup formulation was bought from a US retailer a few months prior to the testing. The brand name of this herbicide was *Roundup Weed & Grass Killer Concentrate Plus*, hereafter “Roundup” or “R”, (Lot I08080/FI/1/5), containing 18 % b.w. glyphosate, 0.73 % diquat-dibromide and, according to label, 81.27 % “other ingredients”. The producer is not required to specify these other ingredients but they are generally thought to consist of mainly water, activator adjuvants and various surfactants (NPIC 2010; PAN 2011; Penner 2000) The Roundup herbicide was produced by Monsanto Company, Marysville, OH, USA. Both chemicals were stored in the dark at room temperature until use.

Prior to the experiments, all clones were acclimatized in the laboratory at the University of Tromsø, Norway. Standardized *D. magna* mother populations brood stock (Benzie 2005) were produced in a fully artificial Aachener Daphnien Medium (ADaM) (modified from Klüttgen et al. 1994) in 4 l glass beakers, fed on a diet of *Selenastrum* sp. green algae. The ADaM medium was produced in de-ionised water using laboratory grade chemicals and evaporated natural sea-salt. The medium was adjusted to a pH of 7.5 (±0.7) with 0.1 M NaOH solution.

Densities of mother-populations brood stock were 20-50 individual mothers in each 4 l beaker. Juveniles less than 24 h old from later than 2nd brood were collected for experiments from these mother populations. All experimental animals were female.

### Definition of variables; acute toxicity testing

For acute toxicity experiments, we used a protocol adapted from “ISO6341 International Standard (ISO1989), United States Environmental Protection Agency OPPTS 850.1010 Daphnid acute toxicity test” (US EPA 1996) and “OECD-202 guidelines for testing of chemicals” from the Organisation for Economic Co-operation and Development (OECD 2004). These guidelines are specific for static acute toxicological testing of hydrophilic chemicals in daphnids. Mixtures of fresh chemicals in ADaM were prepared just prior to each exposure. Following range-finding tests, single series, duplicates or triplicates of at least 5 concentrations were tested for each chemical and *Daphnia* clone. Each experimental unit consisted of 10 juveniles in 100 ml borosilicate glass beakers. Negative controls were established for each experiment and each clone (concentration = 0). Animals were randomly assigned to the different exposure schemes. Subsequent separate testing was performed with juvenile (age <24 h), subadult (age 7 days) and mature (age 19 and 23 days) *D. magna* in the laboratory house-clone (Tromsø-clone), to assess age-dependent susceptibility to glyphosate and Roundup.

Endpoint registered as immobility at 24 and 48 h was recorded by the use of a light-table. Definition of immobility (lack of movement) in guideline OPPTS 850.1010 (US EPA 1996) was used. EC_50_ values for the individual exposure schemes were calculated using probit analysis in SPSS statistical software. Mortality was not observed in any of the negative control groups.

### Definition of variables; chronic toxicity testing

The chronic testing was performed using an extended and modified protocol based on OECD-211 *D. magna* reproduction test guideline (OECD 2008). Juvenile daphnids from brood-stock of the *D. magna* Tromsø-clone were randomly assigned to test regime, kept and reared individually in 100 ml glass beakers. 10 experimental units were set up for each treatment, totalling 110 glasses (animals) for the experiment. Paired test solutions of glyphosate and Roundup were prepared in ADaM medium, as nominal concentrations of 0, 0.05, 0.15, 0.45, 1.35 and 4.05 mg/l of active ingredient (glyphosate). Holding medium was replaced with fresh solutions every third day. All daphnids were fed daily from an algae feed consisting of *Selenastrum* sp. equalling 0.15 mg C/day. Monitoring of survival, reproduction and abortion (counting of visible aborted eggs) was performed every day throughout the 55 day experiment by transferring and observing each glass on a light table.

We quantified the following variables: i) Survival (proportion of surviving animals throughout the life-cycle), ii) Growth (i.e. body size at days 6, 12, 24 and 36), iii) Fecundity (number of live offspring/reproductive day, i.e. in the period between first offspring and end of experiment), iv) Abortion rate (number of visible aborted eggs divided by the sum of juveniles born and the aborted eggs) and v) Offspring body size (length of newborns measured within the first 24 h).

Experimental animals were photographed every 6 days and 1st instar juveniles from their first and second clutch (brood) were photographed within 24 h after birth in order to measure individual body size of mothers and their offspring. Body size was measured from the top of the head to the base of the caudal spine on photographs using ImageJ software. Animals were handled using glass pipettes. Data were analyzed in SPSS, Systat and R-project softwares.

Monitoring of pH, oxygen saturation and laboratory temperature was performed during acclimatization and testing. Oxygen saturation was constantly close to 100 %, pH was within 6.5–8.5 range. Temperature in the laboratory was generally 21 °C (±2). Constant 24/7 uniform artificial lighting was provided from standard fluorescent tubes. All results presented refer to nominal concentrations (mg/l) of the active ingredient (a.i.) glyphosate-IPA in glyhosate and Roundup solutions.

## Results

### Clone- and age-specific acute toxicity

**Fig. 1:**
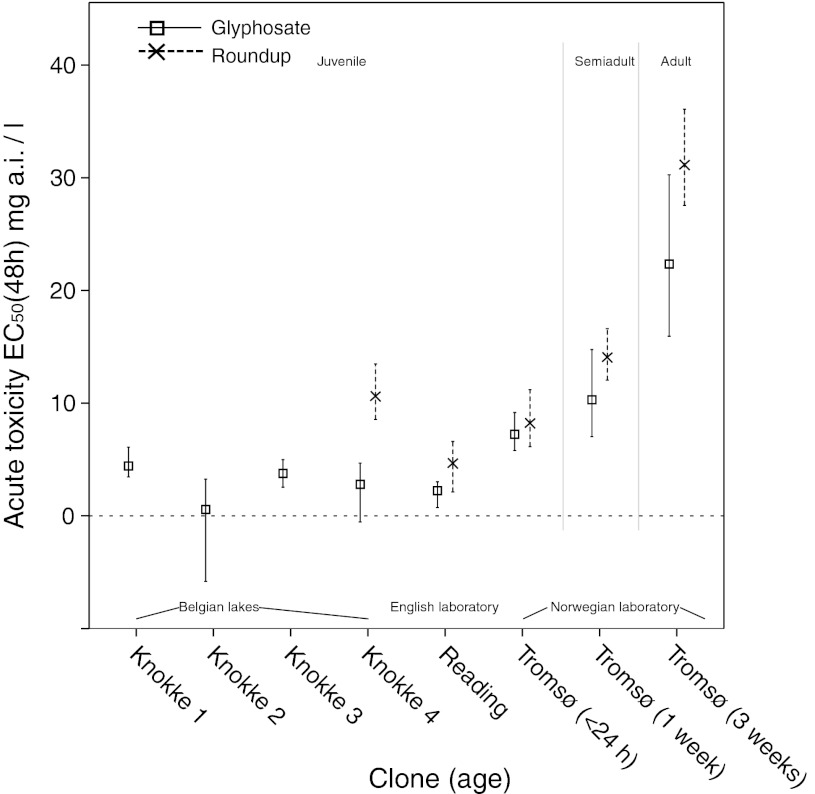
Acute toxicity EC_50_ (48 h) of glyphosate and Roundup to 6 clones and 3 age classes of *D. magna*, calculated as nominal concentrations of active ingredient glyphosate. The clones have different backgrounds, some are wild clones collected in Belgium from pristine lakes (Knokke 1 and Knokke 2), from ponds where some anthropogenic chemicals are expected (Knokke 3) and from ponds in areas of intensive agriculture (Knokke 4). Clones ‘Tromsø’ and ‘Reading’ are from cultures reared for years in laboratories. Error bars denote 95 % CI

Acute toxicity testing of juvenile *D. magna* showed EC_50_ values of 1.4–7.2 mg/l for glyphosate (6 different clones tested) and 3.7–10.6 for Roundup (3 different clones tested). EC_50_ values given for both are content of active ingredient glyphosate (Fig. 1). The differences in clonal sensitivity to glyphosate were rather small, the Tromsø-clone showing lowest susceptibility with an EC_50_ value of 7.2 mg/l. Clonal tolerance to Roundup was generally somewhat higher, but still in the same order of magnitude as for glyphosate. The Knokke 4 clone (KNO 16-F8) showed significantly higher tolerance for Roundup than for glyphosate (Fig. 1). By testing different age classes of the Tromsø-clone an age dependent increase in tolerance was demonstrated. The EC_50_ values of adults were 22 and 31 mg/l for Roundup and glyphosate, respectively (Fig. 1).

### Chronic toxicity

Survival, growth, fecundity, abortion rate and juvenile body size in the 5 test concentrations (0.05, 0.15, 0.45, 1.35 and 4.05 mg/l active ingredient glyphosate), for both glyphosate and Roundup, were compared to the performance of the negative control group to determine effects and levels of no observed effect concentration (NOEC).

### Survival

**Fig. 2:**
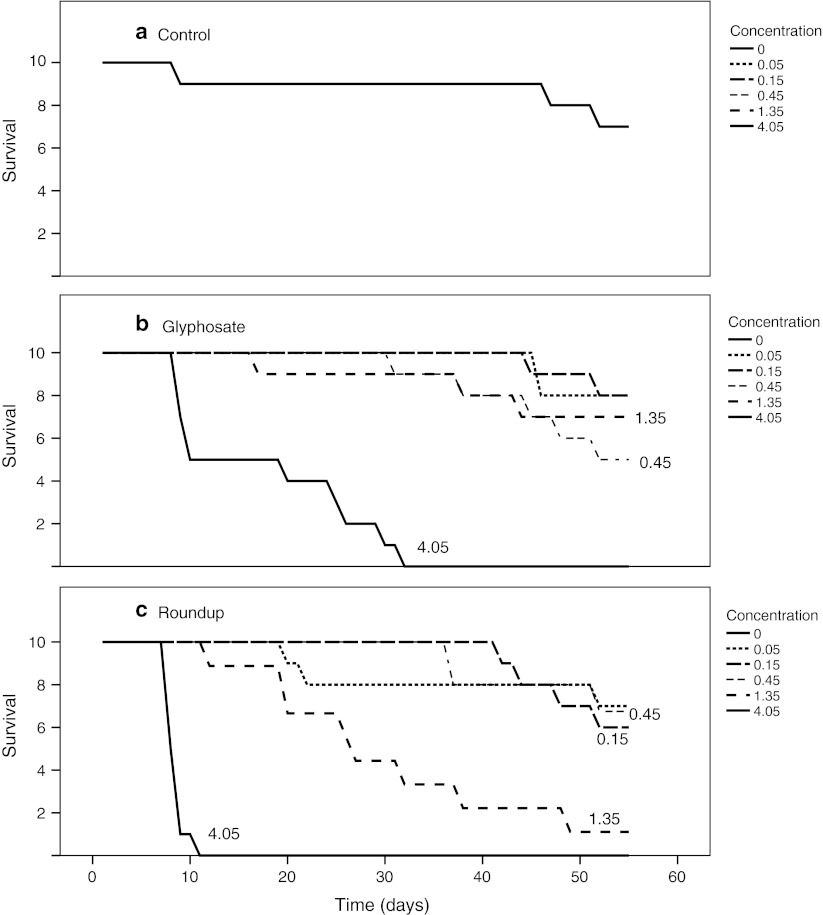
Survival curves of *D. magna* exposed to different concentrations of glyphosate (**b**) and Roundup (**c**). (**a**) shows the negative control

The *D. magna* survival rates were similar for the control and exposed groups for concentrations up to 1.35 mg/l and 0.45 mg/l of glyphosate and Roundup respectively (Fig. 2). At the 4.05 mg/l glyphosate concentration, there was a significant reduction in survival (*p* = 0.0035, CoxPH-test), and the median expected longevity was 15 days. Also at the concentrations 1.35 mg/l and 4.05 mg/l for Roundup a significant reduction in survival (*p* = 0.0055, and *p* = 0.0022, respectively, CoxPH-test) was recorded. Animals exposed to 1.35 and 4.05 mg/l of Roundup had a median expected longevity of 27 and 8.5 days respectively. The NOEC values for glyphosate and Roundup were 1.35 and 0.45 mg/l, respectively.

### Growth

**Fig. 3:**
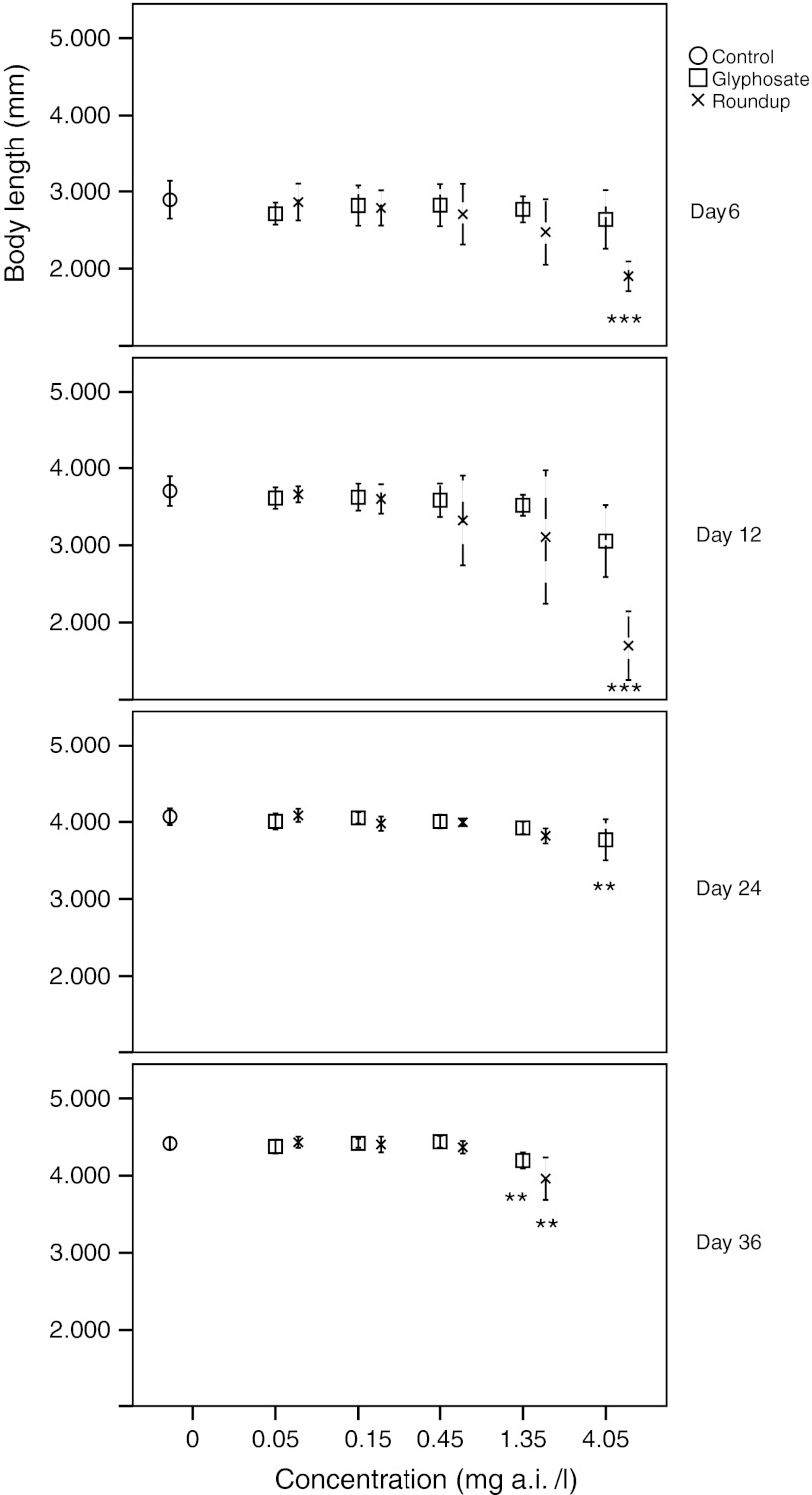
Mean body size at days 6, 12, 24 and 36 of *D. magna* exposed to different concentrations of glyphosate and Roundup. *Error bars* denote 95 % CI and* stars* denote significant difference to the control group, *p < 0.05, **p < 0.01, ***p < 0.001 (ANOVA, Tukey correction)

Length measurements at days 6, 12, 24, and 36 demonstrated reduced growth rates for *D. magna* exposed to glyphosate and Roundup depending on the dose and duration of exposure (Fig. 3). The body sizes of animals exposed to glyphosate concentrations 0.05, 0.15 and 0.45 mg/l as well as to Roundup 0.05, 0.15 and 0.45 mg/l were not significantly different from those of the control group individuals (*p* > 0.05). For glyphosate, significant reduction of body size was found in the 4.05 mg/l concentration at day 24, and in the 1.35 mg/l concentration at day 36. For Roundup, exposure to the 4.05 mg/l concentration entailed a significant reduction in body size at day 6 and at all later time points until the animals were eliminated from analysis by mortality. At 1.35 mg/l animals showed reduced body size at day 36 only. Thus, NOEC levels for growth were 0.45 mg/l for both glyphosate and Roundup.

### Reproduction—fecundity and abortion rate

**Fig. 4:**
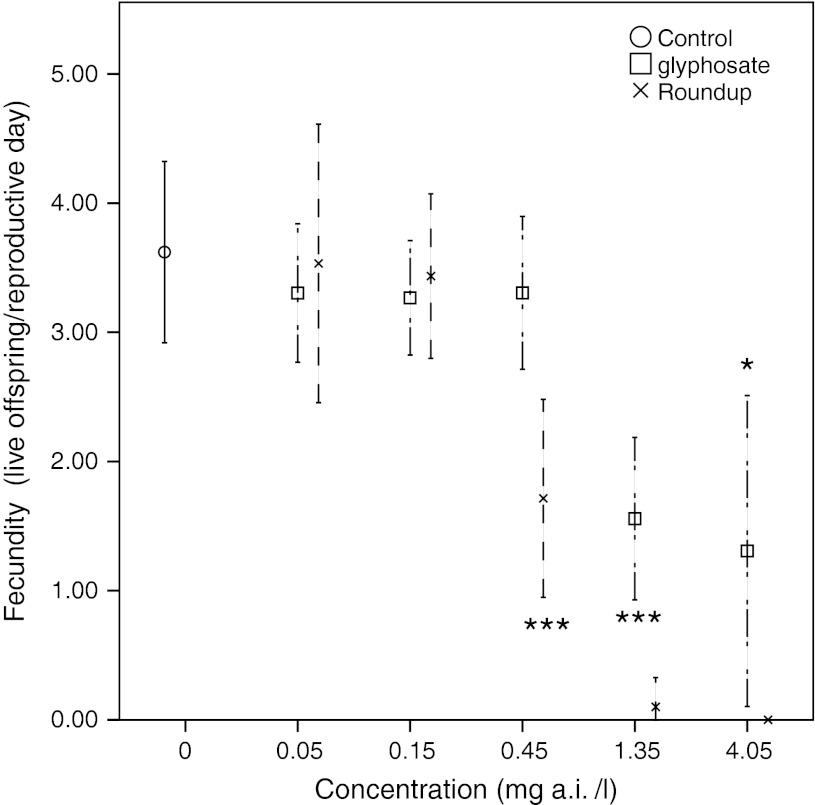
Fecundity as mean number of live offspring/reproductive day for *D. magna* exposed to different concentrations of glyphosate and Roundup. *Error bars* denote 95 % CI and *stars* denote significant difference to the control group, *p < 0.05, **p < 0.01, ***p < 0.001 (ANOVA, Tukey correction)

**Fig. 5:**
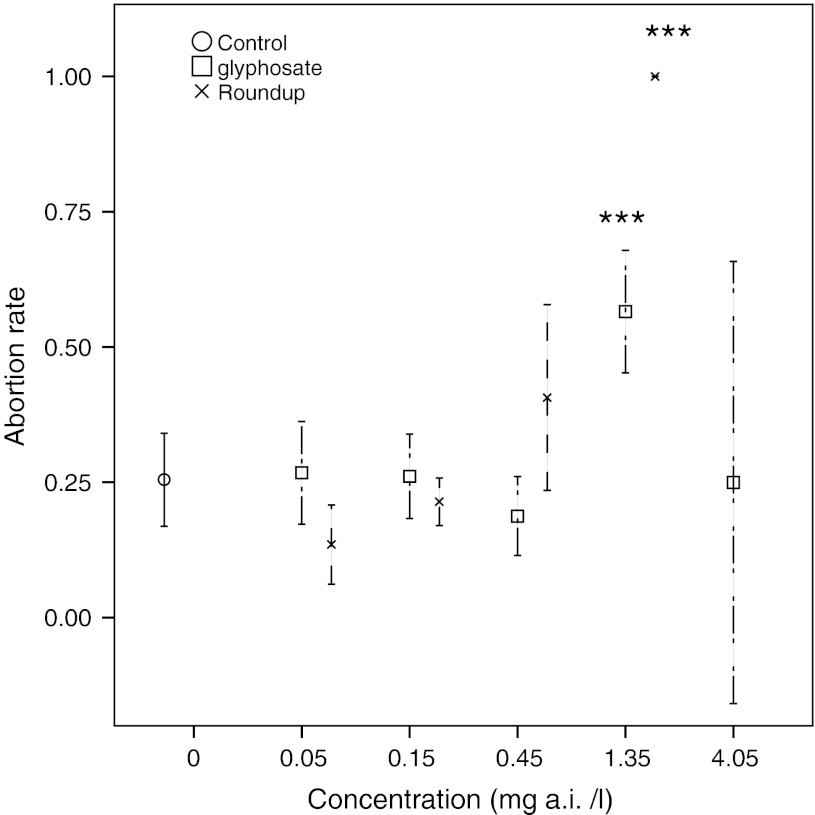
Mean abortion rate of *D. magna* exposed to different concentrations of glyphosate and Roundup. *Error bars* denote 95 % CI and* stars* denote significant difference to the control group, *p < 0.05, **p < 0.01, ***p < 0.001 (ANOVA, Tukey correction)

Exposures to glyphosate concentrations 1.35 and 4.05 mg/l as well as to Roundup concentration 0.45 mg/l significantly decreased fecundity, as compared to the control group (Fig. 4). Animals exposed to 1.35 mg/l of Roundup reached reproductive age, but almost all eggs and developing embryos were aborted (Fig. 5). (Animals exposed to 4.05 mg/l of Roundup died before reaching maturation). Fecundity in animals exposed to glyphosate concentrations 0.05, 0.15, 0.45 and Roundup concentrations 0.05 and 0.15 were not significantly affected. NOEC levels for fecundity were 0.45 mg/l for glyphosate and 0.15 mg/l for Roundup.

The abortion rates for animals exposed to glyphosate as well as Roundup in concentrations of 0.05, 0.15 and 0.45 mg/l, were not significantly different from those of the control group. Abortion rates for animals exposed to 1.35 mg/l of both glyphosate and Roundup were significantly higher than for the control group individuals (Fig. 5), and reached nearly 100 % for animals exposed to Roundup. NOEC levels for abortion were 0.45 mg/l for glyphosate and Roundup.

### Reproduction—size of offspring in first and second clutch

**Fig. 6:**
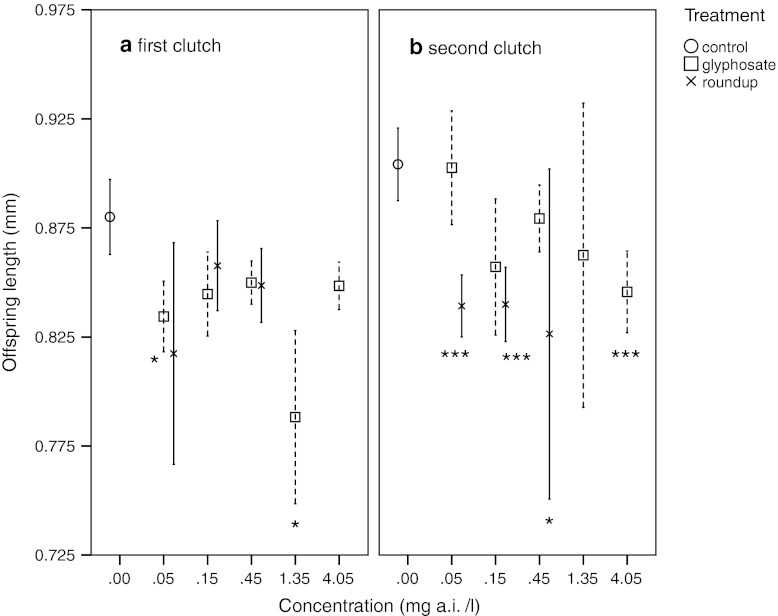
Mean offspring body size in first (**a**) and second (**b**) clutch of *D. magna* exposed to different concentrations of glyphosate and Roundup. *Error bars* denote 95 % CI and *stars* denote significant difference to the control group, *p < 0.05, **p < 0.01, ***p < 0.001 (ANOVA, Tukey correction)

First clutch (brood) juveniles born from groups exposed to 0.05 and 1.35 mg/l glyphosate were significantly smaller than those of the control group. This tendency was not significant in the second clutch, but juveniles born in the 4.05 mg/l concentration were significantly smaller. For Roundup, juveniles from the first clutch were smaller, but differences were not significant. In the second clutch the juveniles from animals exposed to 0.05, 0.15 and 0.45 mg/l Roundup and 4.05 mg/l glyphosate were significantly smaller than those of the control group (Fig. 6).

## Discussion

In the present experiments with *D. magna* we demonstrate that i) glyphosate and Roundup induce EC_50_ at concentrations typically below 10 mg/l in 48 h acute experiments, and ii) chronic exposure, particularly to formulated Roundup, causes serious reproduction damage at levels close to (1.35 mg/l) or even below (0.45 mg/l) accepted threshold values for glyphosate in surface waters in the United States in general (0.7 mg/l) and in the state of California specifically (1.0 mg/l)(California EPA 1997).

### Acute toxicity of glyphosate and Roundup

There were only minor differences in tolerance to acute exposure of glyphosate and Roundup between laboratory clones, clones from natural ponds and clones taken from ponds surrounded by intensive agriculture (Fig. 1). A tendency towards higher tolerance to Roundup was observed, particularly in the Knokke 4-clone. This may be related to this clone’s origin, a pond surrounded by agriculture. Tests of carbaryl pesticide in 10 clones of *D. magna*, two of which were from the same lakes as two of our tested Knokke clones (Knokke 1 and Knokke 4), indicated an overall correlation between land use intensity (farming) and carbaryl tolerance (as EC_50_). These findings were attributed to a genetically based resistance, persistent through several generations of toxicant-free laboratory culturing (Coors et al. 2009). Our observed differences in clonal tolerance of glyphosate toxin can be interpreted as response to environmental differences. However, the biology of this response is unclear at present.

In this work we have shown a relatively uniform susceptibility to glyphosate and Roundup between clones of *D. magna*. This is in contrast to the extreme variation seen between published studies. Accordingly, the highly varying EC_50_ values in *D. magna,* and other species of daphnids reported in printed reviews (Melnichuk et al. 2007a, Pérez et al. 2012, Rico-Martínez et al. 2012), and online databases of pesticide exotoxicology such as the Pesticide Alert Network database (PAN 2011), and the US Environmental Protection Agency Ecotox database (US-EPA 2011), should not be primarily attributed to interclonal differences. *D. magna* toxicity tests are generally considered reproducible and representative, with only small variation between laboratories (Mark and Solbé 1998). Still, test conditions and laboratory environments or other contextual factors may cause the discrepant results. Furthermore, also the *solubility* of the different glyphosate-based chemicals may be a decisive factor in glyphosate toxicity testing of *aquatic* organisms.

In the literature the common name “glyphosate” is used somewhat indiscriminately, including chemical compounds that differ substantially from glyphosate-IPA salt (chemical identity CAS# 38641940), e.g. technical grade glyphosate, which has *low solubility in water* (CAS# 1071836). Toxicological data for technical grade glyphosate are not representative when assessing ecological effects of glyphosate herbicides, which for spraying need to contain a *water soluble* form of glyphosate, e.g. the IPA-salt, as the active ingredient (Dill et al. 2010). During our review of published studies we contacted the authors of 4 papers from groups that had recently published *D. magna* toxicity studies with unspecified glyphosate. These studies were performed in Korea (Le et al. 2010), Turkey (Sarigül and Bekcan 2009), Portugal (Pereira et al. 2009) and Mexico (Dominguez-Cortinas et al. 2008). Authors from 3 of these research groups kindly responded to our information request, confirming that the chemical substance tested was technical grade glyphosate, i.e. the non-soluble version of glyphosate.

Contrary to this, the glyphosate IPA and Roundup formulation tested in the present study is representative for glyphosate herbicides used in agriculture as active ingredient (glyphosate) and formulated product (Roundup). However, variations in toxicity levels may still be expected due to differences in adjuvants and other ingredients of individual formulations (Gasnier et al. 2009, Melnichuk et al. 2007a).

Contrary to the findings of Tsui and Chu (2003) the present work finds acute toxicity of Roundup formulation and active ingredient glyphosate expressed as EC_50_ (48 h) concentrations, to be in the same order of magnitude. This is in accordance with some published work in other aquatic invertebrates such as *Hydra attenuata* (Demetrio et al. 2012).

We have also shown that the *D. magna* tolerance for glyphosate and Roundup is enhanced with increasing age of the animals. This has also been shown for Roundup in other freshwater invertebrates, such as the freshwater shrimp *Caridina nilotica* (Mensah et al. 2011). Both these freshwater invertebrates have relatively low EC_50_-values as adults (22 and 25.3 mg/l for *D. magna* and *C. nilotica*, respectively). Such values are way below previously published results from acute glyphosate toxicity experiments in *D. magna*, even for juveniles. For example, Mcallister and Forbis (1978) presented an EC_50_-value of 759.7 mg/l with a sharp 95 % confidence interval (740.8-779.9).

The European Commission (EC) working document on glyphosate (EC 2002), which forms the basis for European regulation in the context of health and environment, reports the EC_50_ (48) value of 930 mg/l in *D. magna* from Forbis and Boudreau (1981). The authors of the EC paper extrapolate this value into a general EC_50_ value for acute toxicity in aquatic invertebrates. Thus glyphosate is termed “harmless”. According to the 2009 WHO guidelines for pesticide classification (WHO 2009), glyphosate is in class 3; slightly hazardous (in relation to human health). The US EPA has defined glyphosate in Toxicity class 4: “Practically nontoxic”. For a review see Bates (2000).

Furthermore, in 1982 the agrochemicals producer Monsanto presented contrasting data for toxicity of Roundup formulations in *Daphnia sp*., by simultaneously giving LC_50_ (96 h) values of 5.3 mg/l for Roundup and 962 mg/l for glyphosate alone (Servizi et al. 1987). Already in 1979 it was pointed out that technical grade glyphosate had properties (notably reduced water solubility) totally different from those of the glyphosate isopropylamine salt (Folmar et al. 1979). This is, however, an important fact that has been commonly overlooked.

In contrast to other published toxicity data for formulated glyphosate-based herbicides, our results are comparable to those of Folmar et al. (1979) at 3 mg/l for Roundup in *D. magna*, and to 4 of the 6 formulations tested by Melnichuk et al. (2007a) at 4.2–10.2 mg/l. The most recent toxicity data presented by the producer, for the specific brand of Roundup that we have tested, is 11 mg/l EC_50_ (48) for *D. magna* (Monsanto 2011) and thus in accordance with our findings. A recent publication by Sarigül and Bekcan (2009) reports a much higher toxicity of a 48 % commercial Roundup formulation in *D. magna*, with LC_50_ (48 h) values of 0.012 mg/l. We have no explanations for this discrepancy.

Some difference in methods may partly explain published experimental test result variations. For example, Servizi et al. (1987) presented the LC_50_ (96 h)-value for Roundup in *D. pulex* as 25.5 mg/l, but this referred to the Roundup formulation including water. When the authors assessed only the active ingredient glyphosate IPA and the surfactant MONO818 respectively, LC_50_ (96 h)-values of 7.8 and 3.8 mg/l were recorded.

### Chronic toxicity of glyphosate and Roundup

When *D. magna* were exposed to different concentrations in chronic life-cycle experiments, Roundup produced more serious adverse effects than glyphosate alone. This was the case for all tested end-points: survival, growth, fecundity, abortion rates and juvenile body size. Chronic exposure to 0.05 and 0.15 mg/l of Roundup significantly reduced juvenile body size compared to the control group (*p* < 0.001). The same was the case for glyphosate at 0.05 mg/l, but to a lower degree (*p* < 0.05). This is in accordance with findings of Papchenkova (2007) who found juvenile size significantly reduced (*p* < 0.05) by exposure to 2.0 mg/l (6 of 7 generations) and 0.2 mg/l (3 of 7 generations) a.i. concentrations of Roundup.

In our present study no other measured end-points were affected at these concentrations (Table 1). No significant effects on fecundity or abortion rates were seen at concentrations 0.05–0.45 mg/l for glyphosate IPA, but exposure to Roundup at 0.45 mg/l concentration significantly reduced fecundity and increased the abortion rate in addition to the reduced juvenile body size. Following exposure to Roundup at the 1.35 mg/l concentration, significantly impaired survival and growth was observed and reproduction failed completely: all eggs were aborted. A summary of the results from the chronic exposure tests is given in Table 1.

**Table 1:** Observed significant negative effects caused by chronic exposure to glyphosate IPA salt, administered as glyphosate and Roundup in different concentrations, on *D. magna* life-history traits

mg/l a.i. glyphosate IPA	Glyphosate	Roundup
0.05	Reduced juvenile size	Reduced juvenile size
0.15	No observed effect	Reduced juvenile size
0.45	No observed effect	Reduced fecundity
1.35	Reduced growth Reduced fecundity Increased abortion	Reduced growth Increased mortality High abortion
4.05	Reduced growth High mortality Reduced fecundity Increased abortion	High mortality No reproduction

To put these results and concentrations in context: the US EPA general environmental guideline of 0.7 mg/l and the state specific California EPA environmental guideline limit of 1.0 mg/l glyphosate in surface waters are in between the 0.45 and 1.35 mg/l concentrations we use in our tests. The fact that, in the present study, *D. magna* subjected to 1.35 mg/l showed complete reproductive failure, aborting all eggs in early to late stages of embryonic development, indicates that the mentioned environmental guidelines may not be sufficiently restrictive to ensure viable populations of *D. magna* and other aquatic invertebrates.

Ronco et al. (2008) investigated pesticide levels in streams draining several sites with transgenic soybean (glyphosate-tolerant) cultivation in Argentina and found the levels to be; “*often below 1* *mg glyphosate/l, in Arrecifes tributary, although concentration ranges between 1.8 and 10.9* *mg/l (…) were detected*”. The authors concluded that non-target aquatic biodiversity (flora, insects, fish and amphibians) was adversely affected by the pesticide applications.

The levels of glyphosate accepted in surface fresh water vary between nations. As far as we have been able to ascertain, the highest tolerated concentrations are found in the earlier mentioned US-EPA guidelines, 0.7–1.0 mg/l, differing strikingly from the EU guideline limit of 0.0001 mg/l (=0.1 ppb), which seems to be the most restrictive. Canada enforces a limit of 0.065 mg/l (Struger et al. 2008), while Ukraine has set the environmental standard to 0.02 mg/l (Melnichuk et al. 2007a, b).

The results of the few other published studies on chronic exposure of daphnids to glyphosate or glyphosate-based herbicides are distinctly inconsistent.

An industry standard 21-day reproduction test of glyphosate in *D. magna,* based on test concentrations of 0, 26, 50, 96, 186 and 378 mg/l, was additionally reviewed and extrapolated by dr. Wayne C. Faatz, in a March 1983 report to the US EPA (McKee et al. 1982). Neither significant increase of mortality nor reduction of growth was observed in any of the test concentrations. For reproduction, the same report established 50 mg/l as NOEC, a level 100 times higher than the NOEC determined in our experiments.

In contrast, Papchenkova (2007) exposed seven generations of *D. magna* to 0.02, 0.2 and 2 mg/l a.i. glyphosate in Roundup. Significant reduction of endpoints related to fecundity, length of newborn juveniles and growth in first generation was recorded for *D. magna* exposed to a concentration of 2 mg/l. Significant effects on the same endpoints were observed also in subsequent generations for concentrations 2.0 and even 0.2 mg/l, but these effects were not consistent in all measured end-points through all of the 7 generations studied. A follow-up generational study of chronic toxicity in *D. magna* exposed to much higher concentrations of Roundup, i.e. 25 and 50 mg/l a.i. for four generations, showed a significantly reduced fecundity but no adverse effect on the survival of mother animals (Papchenkova et al. 2009).

A similar complexity is evident in a chronic effects study of the Fakel herbicide (48 % a.i. glyphosate IPA) in *Ceriodaphnia affinis* (Melnichuk et al. 2007b). Generational exposures to 10, 5, 2.5, 1 0.1, 0.01 and 0.001 mg/l Fakel established a NOEC of 0.001 mg/l. Even at the very low concentration of 0.01 mg/l, first and second generation fecundity was found to be significantly reduced compared to the control group. Temperature-dependent effects on end-points fecundity and abortion were recorded at test concentrations 1.0–0.1 mg/l. As temperatures were reduced, adverse effects decreased (Melnichuk et al. 2007b). *C. affinis* was shown to be more sensitive to glyphosate herbicide *Fakel* in acute LC_50_ (48) tests than *D. magna* (12.6 mg/l vs. 26.5 mg/l respectively (Melnichuk et al. 2007a).

The acidity of the aquatic environment (or laboratory medium) may also be a relevant factor for the toxicity of glyphosate-based herbicides. Chen et al. (2004) exposed the daphnid *Simocephalus vetulus* to glyphosate herbicide Vision^®^ in sublethal concentrations 0.75 and 1.5 mg/l a.i. (acid equvivalent) under two different pH-regimes (pH 5.5 and 7.5). The authors found that survival, fecundity and juvenile maturation time was affected at both concentrations. The effects were more severe at neutral pH 7.5, versus the lower pH 5.5. Thus, the acidity of the experimental or environmental conditions must be taken into account, in particular when the buffering capacity of the artificial holding medium is low and the toxicants tested are acidic. In our experiments, the variation in acidity spanned a range of 2 pH-units. However, they were still within the pH 6–9 range defined as preferred experimental conditions for *D. magna* testing (OECD 2008).

The term “inert-ingredient” for Roundup formulation additives has been used for product labeling. This is problematic when published literature documents that additives may have significant direct or synergistic toxic effects. Numerous studies have demonstrated that surfactants, often called “adjuvants” or “inert ingredients”, used in Roundup formulations are the primary toxic agents, with toxicity notably higher than glyphosate (“the active ingredient”) alone (e.g. Benachour et al. 2007; Folmar et al. 1979; Gasnier et al. 2009; Melnichuk et al. 2007a).

### Summary and conclusions

According to our experimental work and our literature reviews, we find that the previously published EC_50_ values of 780-930 mg/l for glyphosate (McAllister and Forbis 1978; Forbis and Boudreau 1981) are not representative. The classification of glyphosate as “practically nontoxic” to aquatic invertebrates is based on these non-representative values. The high EC_50_ values have demonstrated tenacious lives, been extensively referred to in the literature and have also found their ways into regulatory documents.

We have found the acute toxicity of glyphosate herbicide active ingredient to be substantially higher, with concentrations below 10 mg/l inducing immobility in *D. magna* within 48 h. If such more conservative EC_50_ values were used, glyphosate would be classified as “toxic” or “moderately toxic” to aquatic invertebrates.

In our chronic studies covering the whole life-cycle of *D. magna*, we demonstrated negative and serious effects at very low concentrations (see Table 1 for a summary), i.e. at levels that can be expected with use of the herbicide Roundup at prescribed dosages in agricultural practice.

The results of our acute and chronic toxicity tests with glyphosate-IPA and Roundup herbicide, in combination with our review of published data, warrant the conclusion that current European Commission and US EPA toxicity classification of these chemicals with regard to effects on *D. magna* and aquatic invertebrates in general, is based on non-representative evidence and needs to be adjusted.
